# The Effects of GABAergic Polarity Changes on Episodic Neural Network Activity in Developing Neural Systems

**DOI:** 10.3389/fncom.2017.00088

**Published:** 2017-09-29

**Authors:** Wilfredo Blanco, Richard Bertram, Joël Tabak

**Affiliations:** ^1^Department of Computer Science, State University of Rio Grande do Norte, Natal, Brazil; ^2^Laboratory of Memory, Sleep and Dreams, Brain Institute, Federal University of Rio Grande do Norte, Natal, Brazil; ^3^Department of Mathematics and Programs in Neuroscience and Molecular Biophysics, Florida State University, Tallahassee, FL, United States; ^4^Institute of Biomedical and Clinical Science, University of Exeter Medical School, Exeter, United Kingdom

**Keywords:** developing neural networks, activity episodes, GABAergic neurons, heterogeneity, excitatory-inhibitory balance

## Abstract

Early in development, neural systems have primarily excitatory coupling, where even GABAergic synapses are excitatory. Many of these systems exhibit spontaneous episodes of activity that have been characterized through both experimental and computational studies. As development progress the neural system goes through many changes, including synaptic remodeling, intrinsic plasticity in the ion channel expression, and a transformation of GABAergic synapses from excitatory to inhibitory. What effect each of these, and other, changes have on the network behavior is hard to know from experimental studies since they all happen in parallel. One advantage of a computational approach is that one has the ability to study developmental changes in isolation. Here, we examine the effects of GABAergic synapse polarity change on the spontaneous activity of both a mean field and a neural network model that has both glutamatergic and GABAergic coupling, representative of a developing neural network. We find some intuitive behavioral changes as the GABAergic neurons go from excitatory to inhibitory, shared by both models, such as a decrease in the duration of episodes. We also find some paradoxical changes in the activity that are only present in the neural network model. In particular, we find that during early development the inter-episode durations become longer on average, while later in development they become shorter. In addressing this unexpected finding, we uncover a priming effect that is particularly important for a small subset of neurons, called the “intermediate neurons.” We characterize these neurons and demonstrate why they are crucial to episode initiation, and why the paradoxical behavioral change result from priming of these neurons. The study illustrates how even arguably the simplest of developmental changes that occurs in neural systems can present non-intuitive behaviors. It also makes predictions about neural network behavioral changes that occur during development that may be observable even in actual neural systems where these changes are convoluted with changes in synaptic connectivity and intrinsic neural plasticity.

## Introduction

Early in development, evidence suggests that neural systems form networks in which synaptic connections are primarily excitatory (see O'Donovan, 1999; Ben-Ari et al., 2007 for reviews). The spontaneous network activity (SNA) is characterized by episodic bursts of intense activity separated by quiescent periods (O'Donovan, 1999; Wenner, 2014), and it is thought that this episodic activity plays essential roles in neural circuit development (Spitzer, 2006; Hanson et al., 2008; Huberman et al., 2008).

One feature of the episodic activity is a strong positive correlation between the duration of an episode and that of the previous inter-episode interval (IEI), but no correlation with the following IEI. This correlation pattern is seen in many tissues, including developing spinal cord (Tabak et al., 2001), developing retina (Sernagor and Grzywacz, 1999), developing cortical networks (Opitz et al., 2002), hyperexcitable hippocampal slices (Staley et al., 1998), disinhibited spinal cord (Rozzo et al., 2002), and spinal cord cultures (Streit, 1993; Streit et al., 2001). In earlier studies, we used mathematical modeling to demonstrate that the episodic behavior, and the striking correlation pattern seen in many developing neural systems, can be explained with a neural network exhibiting activity-dependent synaptic depression (Tabak et al., 2010).

The GABAergic connections that are inhibitory later in development are actually excitatory early in development (Ben-Ari, 2002; Ben-Ari et al., 2007). This is because of developmental differences in the expression level of Cl^−^ co-transporters, with the result that the intracellular Cl^−^ concentration is much higher earlier in development. As a consequence, the Cl^−^ Nernst potential is at a depolarizing value early in development, and later in development shifts to a hyperpolarizing level (Ben-Ari, 2002; Ben-Ari et al., 2007). This transition leads to the establishment of a population of inhibitory neurons that can act as a balance to excitatory neurons in mature neural circuits.

The primary aim of this modeling study is to understand how the developmental transition of GABAergic neurons from excitatory to inhibitory influences the network activity. We ask the question, in a fully connected network that generates episodes of activity when all synapses are excitatory, what happens as the reversal potential for a subset of the synapses is changed from excitatory to inhibitory, replicating the change that occurs during development? This question is difficult to answer experimentally, since other forms of synaptic plasticity (formation and pruning of synapses, and changes in synaptic strength) as well as intrinsic plasticity (changes in ion channel expression) occur simultaneously during development (Wenner, 2014). All of these changes confound the ability to discern the effects of the polarity change in GABAergic synapses.

We perform computer simulations with a network model, but also employ a much simpler mean field model to better understand the effects of the GABAergic synapse polarity change. In many aspects, the two models agree. For example, in both the network and mean field model the strong correlation between episode duration and prior IEI duration is lost as the GABAergic synapses transition from excitatory to inhibitory. However, when the GABAergic synapses become sufficiently inhibitory there is a major disagreement between the results from the network and mean field model. Surprisingly, in this case changes in reversal potential that make the GABAergic synapses more inhibitory actually decrease the intervals between activity episodes in the network, but not the mean field, model. That is, making the connections more inhibitory increases the network activity. We identify a mechanism for this unexpected behavior, as well as conditions under which the behavior does not occur. This involves a group of neurons that are near the borderline for spontaneous activity, and which are similar to the “intermediate neurons” described in previous modeling studies (Tsodyks et al., 2000; Wiedemann and Lüthi, 2003; Vladimirski et al., 2008). Overall, this study illustrates some counterintuitive neural network dynamics that occur when just one element of developmental plasticity takes place. It also illustrates how just a few neurons dictate the fate of the entire population.

## Materials and methods

We simulate the population activity using a mean field model and a neural network model composed of Hodgkin-Huxley-(HH) like neurons with all-to-all coupling. Both models are adapted from Tabak et al. (2010) to include the effects of a GABAergic subpopulation.

### Mean field model

The mean field model contains a variable, *a*, for the activity level of the population and a variable, *s*, for the synaptic efficacy. Both range from 0 to 1, and *a* = 1 means maximum activity, while *s* = 1 means that synaptic coupling is maximal, while lower values of *s* represent some level of synaptic depression. The original formulation of the model, for fully excitatory synaptic coupling (Tabak et al., 2010), is:

(1) $$\tau_{a}\frac{da}{dt}=-a+a_{\infty }\left(wsa-\theta_{0}\right)+n\eta$$

(2) $$\tau_{s}\frac{ds}{dt}=-s+s_{\infty }(a)$$

The equilibrium activity level *a*_∞_ is described by the increasing saturating function $a_{\infty }\left(x\right)=1/\left(1+e^{-x/k_{a}}\right)$, where *k*_*a*_ sets the slope of the sigmoid. The strength of the synaptic coupling is set by parameter *w*, and θ_0_ is a parameter for half activation. The last term in Equation (1) introduces synaptic noise from asynchronous neural firing, with amplitude *n*. At each time step, an independent random number, η, is chosen from a uniform distribution between −0.5 and 0.5. In Equation (2), the steady-state depression level, *s*_∞_, is described by $s_{\infty }\left(a\right)=1/\left(1+e^{\left(a-\theta_{s}\right)/k_{s}}\right)$ with shape parameters θ_*s*_ and *k*_*s*_. Parameter τ_*s*_ is the time constant for the change in *s* in response to a change in *a*.

We modify the original mean field equations to reflect a subpopulation of inhibitory neurons. In the fully excitatory case we did not distinguish between the properties of the excitatory and inhibitory populations, so we continue to assume that the inhibitory neurons have the same properties as the excitatory population. Thus, the effect of decreasing the reversal potential at synapses originating from the inhibitory subpopulation can be described by reducing the synaptic weight parameter in Equation (1). That is,

(3) $$\tau_{a}\frac{da}{dt}=-a+a_{\infty }\left(\left(w-dw\right)sa-\theta_{0}\right)+n\eta$$

where *dw* represents the effects of a decreased reversal potential at a fraction of the synapses. Parameter values are given in Table 1, with *dw* ranging from 0 to 0.19 a.u. For each value of *dw* the simulation was run until either 300 episodes occurred or until *t* > 400,000 a.u. We note that this is identical to a model including *a* and *s* variables for the excitatory population and a separate model with *a* and *s* variables for the inhibitory population, if one assumes that the cell types in the population are identical and if *a* and *s* variables start with the same initial conditions in the two populations. The equations are solved numerically using the Euler-Maruyama method with Δ*t* = 0.01.

**Table 1:** Parameters of the mean field model (arbitrary units).

**Parameter**	**Description**	**Value**
*w*	Excitatory synaptic weight	0.8
*dw*	Change in synaptic weight	0.0–0.19
θ_*0*_	Half activation	0.17
*k_*a*_*	*a*_∞_ slope parameter	0.05
θ_*s*_	Half depression	0.2
*k_*s*_*	*s*_∞_ slope parameter	0.05
*n*	Noise amplitude	0.5
τ_*s*_	Time constant	250
τ_*a*_	Time constant	1

### Neural network model

The neural network model consists of a population of HH like neurons with all-to-all coupling, as in Tabak et al. (2010). Heterogeneity is established in the population by randomly picking the applied current parameter, *I*_*app*_, from a uniform distribution over the range −10 to 5 μA/cm^2^. This same set of applied currents is used for each value of the GABA synapse reversal potential. Each model neuron is described by four variables, the first two are the membrane potential (*V*) and the fraction of activated delayed rectifier K^+^ channels (*n*), with the following differential equations:

(4) $$C\frac{dV_{j}}{dt}=-\left[I_{Na_{j}}+I_{K_{j}}+I_{l_{j}}+I_{syn,e_{j}}+I_{syn,i_{j}}-I_{app_{j}}\right]$$

(5) $$\frac{dn_{j}}{dt}=\alpha_{n}\left(V_{j}\right)\left(1-n_{j}\right)-\beta_{n}(V_{j})n_{j}$$

where the Na^+^ current is simplified and assumes instantaneous activation as in Rinzel (1985):

(6) $$I_{Na_{j}}=g_{Na}m_{\infty }^{3}(V_{j})(0.8-n_{j})(V_{j}-V_{Na})$$

The K^+^ and leakage currents are, respectively:

(7) $$I_{K_{j}}=g_{K}n_{j}^{4}\left(V_{j}-V_{K}\right)$$

(8) $$I_{l_{j}}=g_{l}(V_{j}-V_{l})$$

A subset of the *N* neurons in the population are inhibitory (*N*_*i*_ neurons), while the remainder are excitatory (*N*_*e*_ neurons). We assume that both types of neuron experience activity-dependent synaptic depression. The synaptic currents have the form

(9) $$I_{syn,e_{j}}=g_{syn,e_{j}}(V_{j}-V_{exc})$$

(10) $$I_{syn,i_{j}}=g_{syn,i_{j}}(V_{j}-V_{inh})$$

where the excitatory and inhibitory synaptic conductances depend on the activity of the presynaptic neuron (*a*_*j*_) and its level of efficacy (*s*_*j*_). If *a*_*j*_ = 1 then the neuron fully activates post-synaptic currents onto its targets, and if *s*_*j*_ = 1 then these currents exhibit no depression. The conductances are:

(11) $$g_{syn,e_{k}}=\frac{{\bar{g}}_{syn}}{N}\left(\sum_{j}^{N_{e}}a_{j}s_{j}-a_{k}s_{k}\right)$$

(12) $$g_{syn,i_{k}}=\frac{{\bar{g}}_{syn}}{N}\left(\sum_{j}^{N_{i}}a_{j}s_{j}-a_{k}s_{k}\right)$$

where for *g*_*syn*,*e*_*k*__ the summation is over excitatory neurons, and for *g*_*syn*,*i*_*k*__ it is over inhibitory neurons. The parameter *ḡ*_*syn*_ is the synaptic conductance of an active synapse (*a*_*j*_ = 1) with full efficacy (*s*_*j*_ = 1). The sums are over all neurons except for the postsynaptic neuron itself (neurons do not synapse onto themselves). For each of the *N* neurons, for the neuron activity *a*_*j*_ and efficacy *s*_*j*_ are described by:

(13) $$\frac{da_{j}}{dt}=\Pi \left(V_{j}\right)\alpha_{a}\left(1-a_{j}\right)-\beta_{a}a_{j}$$

(14) $$\frac{ds_{j}}{dt}=\alpha_{s}\left(1-s_{j}\right)-\Pi (V_{j})\beta_{s}s_{j}$$

The step-like function $\Pi \left(V_{j}\right)=1/\left(1+e^{\left(v_{th}-V_{j}\right)/kv_{j}}\right)$ reflects synaptic release when the presynaptic voltage *V*_*j*_ depolarizes above *V*_*th*_ during an action potential. When this occurs, Π(*V*_*j*_) goes from ≈0 to ≈1. Both Equations (13) and (14) have the form of a first-order reaction, where the *a*_*j*_ activation rate (α_*a*_) is multiplied by Π(*V*_*j*_), as is the *s*_*j*_ depression rate (β_*s*_). Thus, a presynaptic action potential causes *a*_*j*_ to increase and *s*_*j*_ to decrease. The average network activity and synaptic efficacy are 〈*a*〉 $=\frac{1}{N}\sum_{j=1}^{N}a_{j}$ and 〈*s*〉 $=\frac{1}{N}\sum_{j=1}^{N}s_{j}$, respectively. The change of polarity that occurs in GABAergic synapses during development is simulated by decreasing the reversal potential values (*V*_*inh*_) of the inhibitory neurons, starting from 10 mV and ranging to −72 mV in increments of 2 mV. Network model parameters are shown in Table 2.

**Table 2:** Parameters of the network model using Hodgkin-Huxley-type neurons.

**Parameter**	**Description**	**Value**
*g_*l*_*	Leak conductance	0.1 mS/cm^2^
*V_*l*_*	Leak reversal potential	−49.4 mV
*g_*Na*_*	Sodium conductance	36 mS/cm^2^
*V_*Na*_*	Sodium reversal potential	55 mV
*g_*k*_*	Potassium conductance	12 mS/cm^2^
*V_*k*_*	Potassium reversal potential	−72 mV
*ḡ*_*syn*_	Max. synaptic conductance	3.6 mS/cm^2^
*V_*exc*_*	Excitatory reversal potential	10 mV
*V_*inh*_*	Inhibitory reversal potential	10 to −72 mV
*I_*app*_*	Input or applied current	−10 to 5 μA/cm^2^
α_*a*_	Synaptic activation rate	1 ms^−1^
β_*a*_	Synaptic decay rate	0.1 ms^−1^
α_*s*_	Synaptic recovery rate	0.0015 ms^−1^
β_*s*_	Synaptic depression rate	0.12 ms^−1^
*V_*th*_*	Threshold for activation/depression	−20 mV

Voltage is in mV, time is in ms, and the capacitance, conductance, and currents are normalized with respect to surface area (with *C* = 1 μF/cm^2^). Results are presented for *N* = 100 neurons in a recurrent network (all-to-all coupling), with *N*_*e*_ = 80 excitatory neurons and *N*_*i*_ = 20 inhibitory neurons. Simulations are run until either 200 episodes are produced or 1,000 s of simulation time.

The software development framework was Eclipse IDE for C/C++ using the MinGW compiler. The Boost C++ library (Schling, 2011), specifically Boost.Numeric.Odeint, was used to solve the ordinary differential equations using a fourth-order Runge-Kutta algorithm with time step of 0.01 ms. Simulations with *N* = 200–300 neurons gave qualitatively similar results, and are shown in Supplemental Material. The source code was tested on Windows and Linux platforms, and the files are available as freeware at www.math.fsu.edu/~bertram/software/neuron.

### Episode detection

Episodes were detected by monitoring the population activity level *a* in the mean field model and the mean activity level, <*a*>, in the network model. Initial transients were not included in the analysis. The absolute minimum (*minA*) and maximum (*maxA*) values of activity were determined and the maximum rate of change of the activity (*slopemax*) was also determined. An episode was identified when the mean activity increased by more than 0.17Δ*A* and with a rate of change greater than 0.25·*slopemax*. The end of an episode is identified when the mean activity drops by more than 0.17Δ*A*. Similar event detection criteria were used in Fletcher et al. (2016).

## Results

The goal of this study is to determine how a polarity change from excitatory to inhibitory in the GABAergic synapse reversal potential influences the episodes of activity found in developing neural networks. Changes in synaptic connectivity are not considered, so as to focus just on the polarity change. We use two models to do this, as described in Materials and Methods. One is a two-variable mean field model for population activity, while the other is a neural network model with all-to-all coupling, assuming that 20% of the 100 neurons are GABAergic. We begin with an analysis of the mean field model.

### Making GABAergic neurons inhibitory increases the time between episodes in the mean field model

The mean field model (Equations 1, 2) describes the mean firing rate or activity (*a*) of a homogeneous population of neurons, as well as a synaptic efficacy variable (*s*). Both variables range from 0 to 1, with one meaning maximum activity/efficacy. We modified the model of Tabak et al. (2010) by introducing a parameter *dw* that reflects inhibitory connections. The change of polarity from excitatory to inhibitory is then simulated by increasing *dw*, effectively reducing the overall connectivity. This is a very simple representation of the neural network and the effects of polarity change in the GABAergic synapses, but it does reveal some properties that are shared by the more complex neural network model. It has the desirable property that its simplicity facilitates analysis.

**Figure 1:**
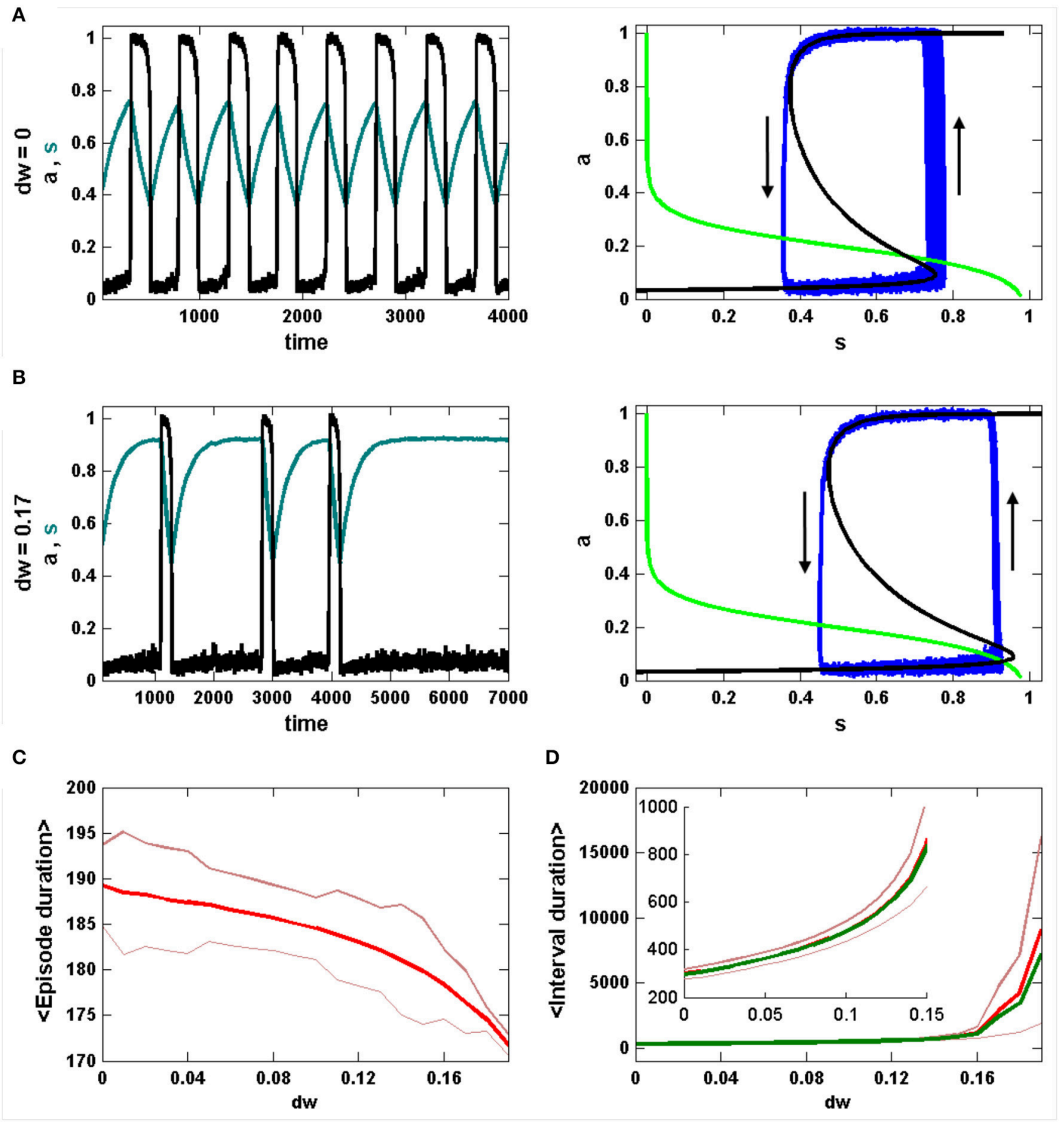
A snapshot of spontaneous episodic activity features over a range of inhibitory weight (*dw*) values in the mean field model. **(A)** Spontaneous episodic activity (*a*, black curve) and synaptic efficacy (*s*, dark green curve) and the trajectory in the phase plane (blue curve, right panel) with *dw* = 0. The *s*- and *a*-nullclines (with no noise, *n* = 0) are superimposed. The *s*-nullcline is in green and the *a*-nullcline is in black. **(B)** With *dw* = 0.17 the episodes are shorter, the intervals are longer, and there is less variability in the values of *s* where an episode begins. **(C)** The mean episode duration (bold red curve) declines monotonically with *dw*. Thin red curves are mean ± standard error. **(D)** Interval durations increase slowly with *dw* until *dw* = 0.17, beyond which the increase in interval duration is much more extreme and the mean (thick red curve) and median (thick green curve) begin to diverge. The thin red curves are the mean ± standard error.

Figure 1A shows the dynamics of activity and efficacy when there is no inhibition (*dw* = 0). The left panel shows episodes of activity with period of ~500 a.u. (black curve). During each episode the synaptic efficacy declines (green curve), reflecting synaptic depression that slowly degrades the strength of the autofeedback of *a* onto itself. This ultimately results in termination of an episode and a drop in activity to near 0. The efficacy variable now slowly increases, reflecting recovery from synaptic depression. This ultimately results in the start of a new episode and the cycle begins again. Indeed, this is a limit cycle oscillation, with small perturbations due to the effects of noise (the *n*η term in Equation 3). The right panel shows several cycles in the *s-a* phase plane, along with *s*- and *a*-nullclines (green and black curves, respectively). The trajectory moves along the top and bottom branches of the *a*-nullcline as a relaxation oscillation, reflecting the much faster dynamics of *a* relative to *s*. The beginning of an episode occurs near *s* = 0.75, when there is a jump from the down state to the up state. Notice that there is a great deal of variability in the *s*-value of this jump, due to the effects of noise occurring in the vicinity of the lower knee of the *a*-nullcline (see Tabak et al., 2010 for details). In contrast, there is little variability in the value of *s* where episodes are terminated (*s* ≈ 0.35). We return to this shortly.

Figure 1B shows the behavior in the presence of significant inhibition (*dw* = 0.17). The oscillation period is now much larger, due entirely to an increase in the IEI. In fact, the periodic occurrence of episodes is no longer a limit cycle oscillation. Instead, the deterministic system (with *n* = 0 in Equation 3) has a stable equilibrium (where the nullclines intersect) on the lower branch of the cubic *a*-nullcline, corresponding to a system at rest. The episodes occur as a result of the noise, which occasionally perturbs the system past the episode threshold. In the phase plane (Figure 1B, right), this is reflected in very little variation in the *s*-value where episodes are initiated (*s* ≈ 0.93); prior to each episode the phase point is stuck at the same location until an appropriate perturbation kicks it over the threshold (the middle branch of the *a*-nullcline).

Statistics for the episode and inter-episode duration over a range of inhibition levels are shown in Figures 1C,D. As the inhibition increases the mean episode duration decreases (Figure 1C). The standard deviation also declines with *dw*, and there is a reduction in the coefficient of variation. In contrast, the IEI increases as *dw* is increased (inset), and the rate of increase grows sharply at *dw* ≈ 0.17 when the production of episodes bifurcates from limit cycle oscillations to noise-induced phenomena. Also at this bifurcation point, the standard deviation grows quickly since the episode is triggered entirely by the noise, and the mean IEI and median IEI begin to diverge.

**Figure 2:**
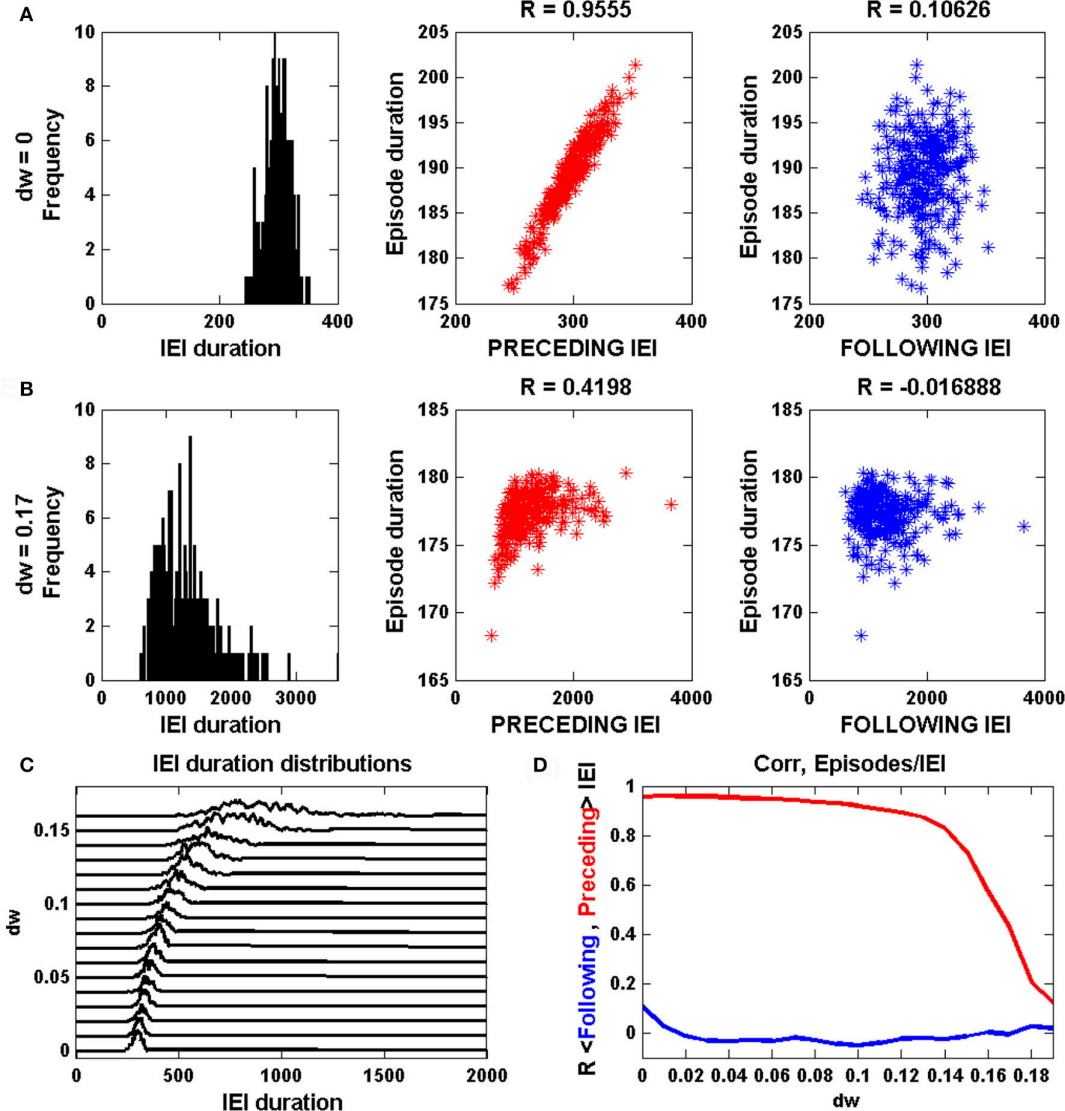
Analysis of episode durations and inter-episode interval (IEI) durations generated by the mean field model. **(A)** Without inhibition (*dw* = 0) the IEI durations have a unimodal distribution and the episode duration is strongly correlated with the preceding IEI, but not the following IEI. **(B)** With inhibition (*dw* = 0.17) the IEI durations have a long tail at higher values and the correlation between episode duration and preceding IEI duration is lost. **(C)** The IEI duration distributions are shown together for a range of values of *dw*. The peak of the distribution moves rightward and the distribution spreads out for larger *dw*. **(D)** The correlation between episode duration and preceding IEI duration (red) drops sharply beyond *dw* = 0.14. There is never a correlation between episode duration and the following IEI duration (blue).

The episode statistics are viewed in a different way in Figure 2. Without inhibition, the IEI distribution is roughly Gaussian (Figure 2A, left). When each episode duration is plotted against the prior IEI duration there is a clear positive linear correlation; a longer IEI results in a longer next episode (Figure 2A, middle). As described in detail in Tabak et al. (2010), this reflects the fact that the effect of noise is stronger during the IEI, and it acts on the limit cycle oscillation primarily by perturbing the phase point into a new episode at random points around the lower knee of the *a*-nullcline. If the jump occurs before the lower knee, the phase point then has less distance to cover to reach the upper knee that terminates an episode. Thus, short IEIs result in short episodes. However, a short episode does not result in a short next IEI, since the noise rarely terminates an episode before the phase point reaches the upper knee of the *a*-nullcline; thus, IEIs almost always start from the same value of *s* and there is no correlation between the episode duration and the following IEI (Figure 2A, right).

Things change dramatically when the strength of the inhibition is sufficient to terminate the limit cycle oscillations. In Figure 2B, with *dw* = 0.17, the IEI duration distribution is no longer Gaussian, but instead has a large tail for larger durations. It is this tail that causes the deviation between the mean and median of the distribution. Furthermore, the correlation between episode duration and the prior IEI is lost (Figure 2B, middle), since now all episodes start at roughly the same values of *s* and therefore the phase point travels about the same distance along the upper branch of the *a*-nullcline for each episode. As before, there is no correlation between episode duration and that of the next IEI (Figure 2B, right).

The change of the IEI distribution over a range of inhibition levels (*dw* from 0 to 0.19) is shown in Figure 2C. Each curve shows the IEI distribution for the corresponding value of *dw*. It is clear that as *dw* is progressively increased the peak of the distribution shifts rightward to longer IEI durations and the distribution spreads out. Thus, the timing of the episodes becomes much more irregular as the synaptic coupling is decreased. Figure 2D summarizes the correlation information for the same range of *dw* values. Up until *dw* ≈ 0.14 there is a strong positive correlation between episode durations and the previous IEI durations, but this drops off sharply for the largest levels of inhibition, reflecting the bifurcation from noisy limit cycle behavior to noise-induced episodes.

In summary, the mean field model tells us that as the self-coupling is weakened the activity episodes get shorter and the IEI durations get larger. Also, the IEI distribution becomes skewed with a tail for longer durations, and the correlation between episode duration and the previous IEI declines. Near a bifurcation point it drops quickly to 0. We next investigate whether these characteristics are present in the more complex (and more biological) neural network model, and also whether additional behaviors are observed.

### Making GABAergic neurons inhibitory in the neural network model has paradoxical effects

For the neural network model we employ HH-like model neurons exhibiting all-to-all coupling (see Materials and Methods). Out of the 100 neurons in the network, 20 are considered GABAergic. Unlike the mean field model, the system is now purely deterministic, but it is heterogeneous in that the applied current parameter, *I*_*app*_, is uniformly distributed among the 100 neurons over the interval −10 to 5 μA/cm^2^. Of these 100 neurons, an average of 10 are spontaneously active in the absence of synaptic input. Each neuron *j* has a synaptic drive variable, *a*_*j*_, that increases when the neuron is spiking. There is also a synaptic efficacy variable for each neuron, *s*_*j*_, that slowly declines when the neuron is spiking and that reflects synaptic depression.

**Figure 3:**
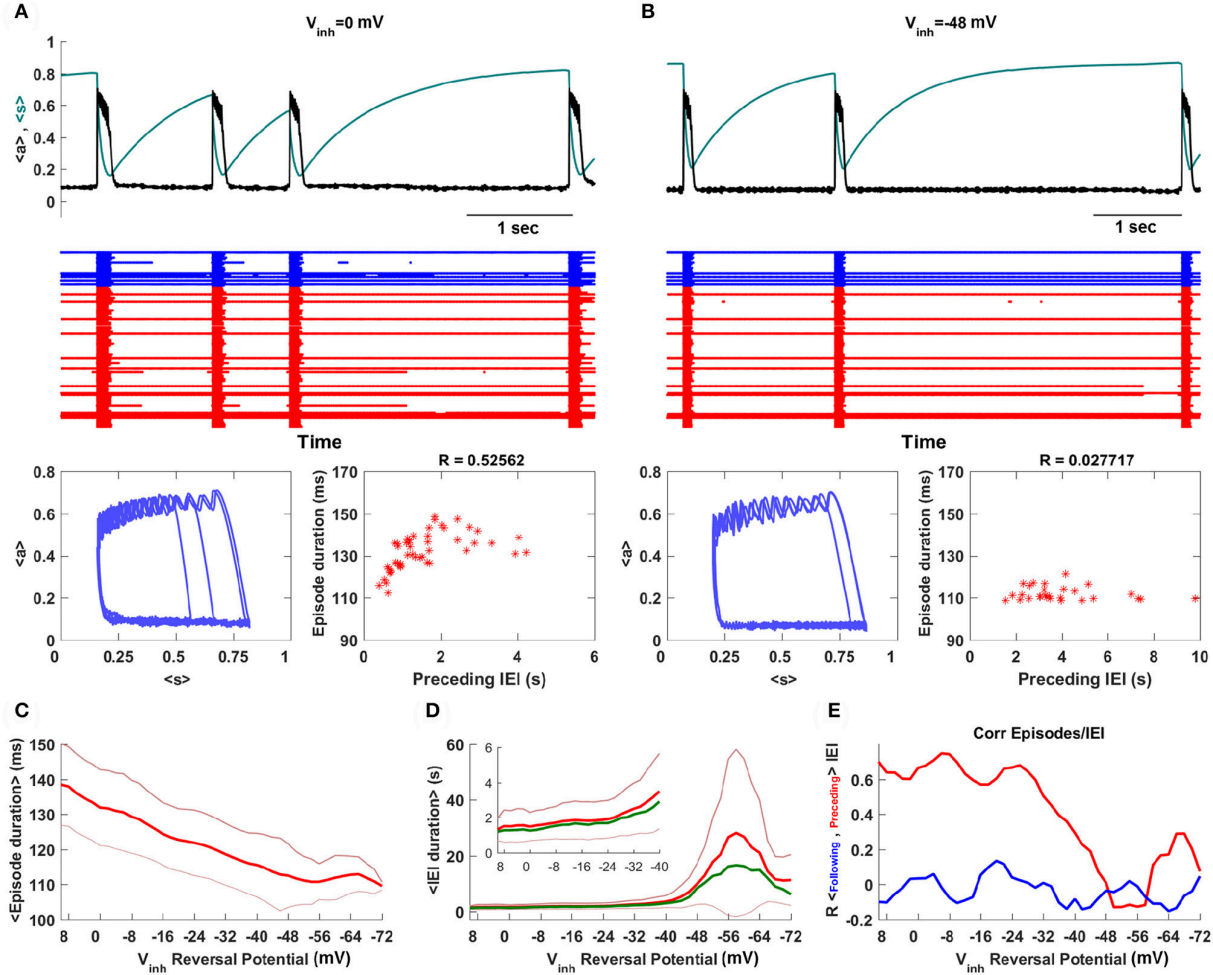
Spontaneous episodic activity of the neural network model with 100 neurons, of which 20 are GABAergic, and all-to-all coupling. **(A)** A snapshot of the episodic activity, showing the population-mean synaptic activity <*a*> and efficacy <*s*>. In the raster plot the blue traces correspond to GABAergic neurons, while the red traces correspond to glutamatergic neurons. In this simulation *V*_*inh*_ = 0 mV, corresponding to early development when GABAergic synapses are excitatory. **(B)** Later in development the GABAergic neurons become inhibitory, as in this case with *V*_*inh*_ = −48 mV. There is much less variation in 〈*s*〉 at the start of an episode, and no correlation between the durations of episodes and the preceding IEI. **(C)** The mean episode duration declines as the synaptic reversal potential of GABAergic neurons is changed from excitatory to inhibitory. The thick red curve is the mean and the thin red curves are mean plus/minus standard deviation. **(D)** As expected, the mean IEI duration increases as the GABAergic synapses are made more inhibitory, but unexpectedly it reaches a maximum and then declines with greater inhibition. In addition to mean and standard deviation, the median (thick green curve) is also plotted. **(E)** The correlation between episode duration and preceding IEI declines as GABAergic neurons become inhibitory, and there is never a correlation between episode duration and the following IEI duration.

Figure 3A shows episodic behavior of the network when all synapses, including GABAergic synapses, are excitatory. The population-averaged activity, <*a*>, is low between episodes, but rises rapidly during the start of an episode. Episode termination is also rapid, reflected by a sharp drop in <*a*>. Also shown is the population-averaged synaptic efficacy, <*s*>, which slowly increases between episodes and more rapidly declines during an episode, reflecting synaptic depression across the network. A raster plot shows the activity of all 100 neurons in the network over 5 s of time. The red traces show the activity of glutamatergic neurons, while the blue traces show the activity of GABAergic neurons. It is evident that some neurons spike continuously, some are only active during episodes, while the remaining neurons are often active immediately before and during episodes and for some time after episodes. These latter neurons therefore seem to correspond to what have been termed the “intermediate population” in a prior study (Vladimirski et al., 2008). They are important as the initiators of episodes; all other neurons are firing already or only fire once the episode is initiated. When <*a*> and <*s*> are plotted together, below the raster plots, the cyclic behavior has a somewhat similar appearance to that of the mean field model in limit cycle mode (Figure 1A). That is, there is little variation in the value of <*s*> at the termination of an episode, but a great deal of variation in <*s*> at the start of an episode. The variation is now due to network heterogeneity rather than intrinsic noise, but the mechanism is essentially the same. This is confirmed by the linear correlation between the duration of the previous IEI and the episode duration (sample size of 50 episodes).

Simulations with the network model reveal that reducing the synaptic coupling strength has effects that are very similar to those of the mean field model. Thus, if *ḡ*_*syn*_ is decreased the inter-episode duration increases, as in Figure 2B. However, a more accurate way to simulate the developmental change in GABAergic synapse polarity is simply to reduce the synaptic reversal potential for the GABAergic neuron population (something that could not be done with the mean field model). This is the approach that we take next.

When the reversal potential of the GABAergic population is decreased to *V*_*inh*_ = −48 mV there are fewer episodes due to longer IEIs (Figure 3B). Also, there is considerably less variability in the *s* value for episode initiation and a loss in correlation between episode duration and the previous IEI. This is all in agreement with expectation established by the mean field model (Figures 1B, 2B). This is quantified over a range of inhibitory reversal potentials in Figures 3C–E. In agreement with the mean field model, as *V*_*inh*_ is reduced from 10 to −72 mV, making GABAergic neurons progressively more inhibitory, the mean episode duration declines (Figure 3C). Also as expected, the mean IEI initially increases as *V*_*inh*_ is reduced and the mean and median begin to diverge by about *V*_*inh*_ = −48 mV (Figure 3D), consistent with the mean field model. Associated with this divergence is a drop in the correlation between the episode durations and the previous IEI durations (Figure 3E). There is a surprise however: for values of *V*_*inh*_ less than about −58 mV the mean IEI declines when *V*_*inh*_ is further reduced. Why does making the GABAergic synapses more inhibitory make the IEIs shorter? This behavior was not observed in the mean field model, and in fact it is not at all clear why it should happen here. We investigate this paradoxical behavior next.

### System priming is responsible for the unexpected decline in the inter-episode interval duration

**Figure 4:**
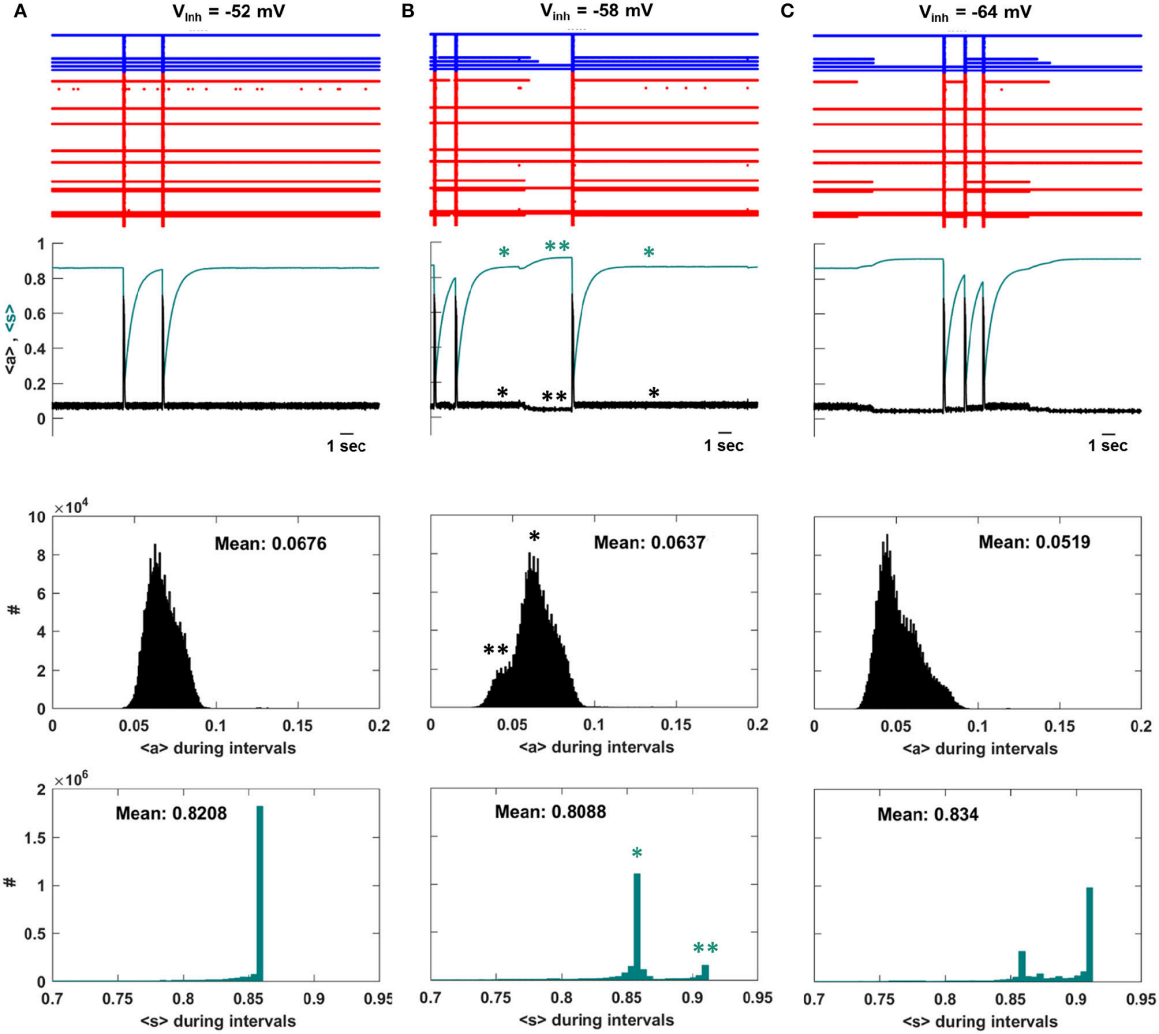
Activity at three values of *V*_*inh*_. **(A)** To the left of the IEI duration peak, *V*_*inh*_ = −52 mV, the intermediate neurons mostly continue to fire during intervals and an episode is sometimes initiated when an intermediate neuron fires. As a result, 〈*s*〉 is flat during the intervals and there is no priming. There is a single large peak in the distribution of 〈*s*〉 during the intervals. **(B)** At the peak of the IEI duration distribution, *V*_*inh*_ = −58 mV, some of the intermediate neurons turn off during intervals (^**^), causing 〈*s*〉 to rise and priming the system for a new episode. There are now two peaks in the distribution of 〈*s*〉 during the intervals, corresponding to primed (^**^) and unprimed (^*^) states of the network. **(C)** To the right of the IEI duration peak, *V*_*inh*_ = −64 mV, the priming effect is accentuated. The activity during the IEIs is now lower and as a consequence the primed peak in the 〈*s*〉 distribution is now dominant.

To understand the decline in IEI duration that occurs for *V*_*inh*_ < −58 mV we examine the system dynamics for values of *V*_*inh*_ at the peak of the IEI duration histogram and on either side of it. Figure 4A shows several seconds of network activity to the left of the peak, *V*_*inh*_ = −52 mV. Most of the neurons are silent between episodes, while about 10 spike continuously. There is a single intermediate neuron that turns on and off several times between episodes, and on some occasions this neuron is the trigger for an episode. During IEIs the average activity, <*a*>, is mostly low, while the mean efficacy, <*s*>, is mostly high since the majority of the neurons are quiescent and their efferent synapses are therefore not depressed. This is shown in the bottom two histograms, which give the population-mean values at time points throughout the simulation. Figure 4B shows activity at the peak of the IEI duration histogram, with *V*_*inh*_ = −58 mV. Because of the increased inhibition, some of the neurons that were previously active during the IEIs now often turn off at some time between episodes. When this occurs, <*a*> declines somewhat and <*s*> increases (second panel, ^**^). Thus, the mean activity of the network decreases, but the mean synaptic efficacy increases. We refer to this increase in mean connectivity strength as “priming.” The decline in <*a*> and the rise in <*s*> are quantified as histograms in the lower two panels. The single star (^*^) indicates the state of the system when not primed, while the double star (^**^) indicates the state of the system during a priming event. There is a left shoulder in the <*a*> inter-episode histogram and a second peak in the <s> histogram reflecting the priming, which is a relatively rare event.

Priming becomes more prevalent as the GABAergic neurons are made more inhibitory. With *V*_*inh*_ = −64 mV the intermediate neurons are off during most of the inter-episode periods, so that the small shoulder in <*a*> and small peak in <*s*> that were present at *V*_*inh*_ = −58 mV now dominate the histograms (Figure 4C). As a consequence, the mean IEI activity is typically lower than at *V*_*inh*_ = −58 mV, and the mean efficacy during the IEI is typically higher. When the efficacy is higher, it is more likely that activation of a single neuron will trigger an episode. In the next section we provide further evidence that the priming effect is due to the intermediate neurons.

### A small number of intermediate neurons control the network behavior

**Figure 5:**
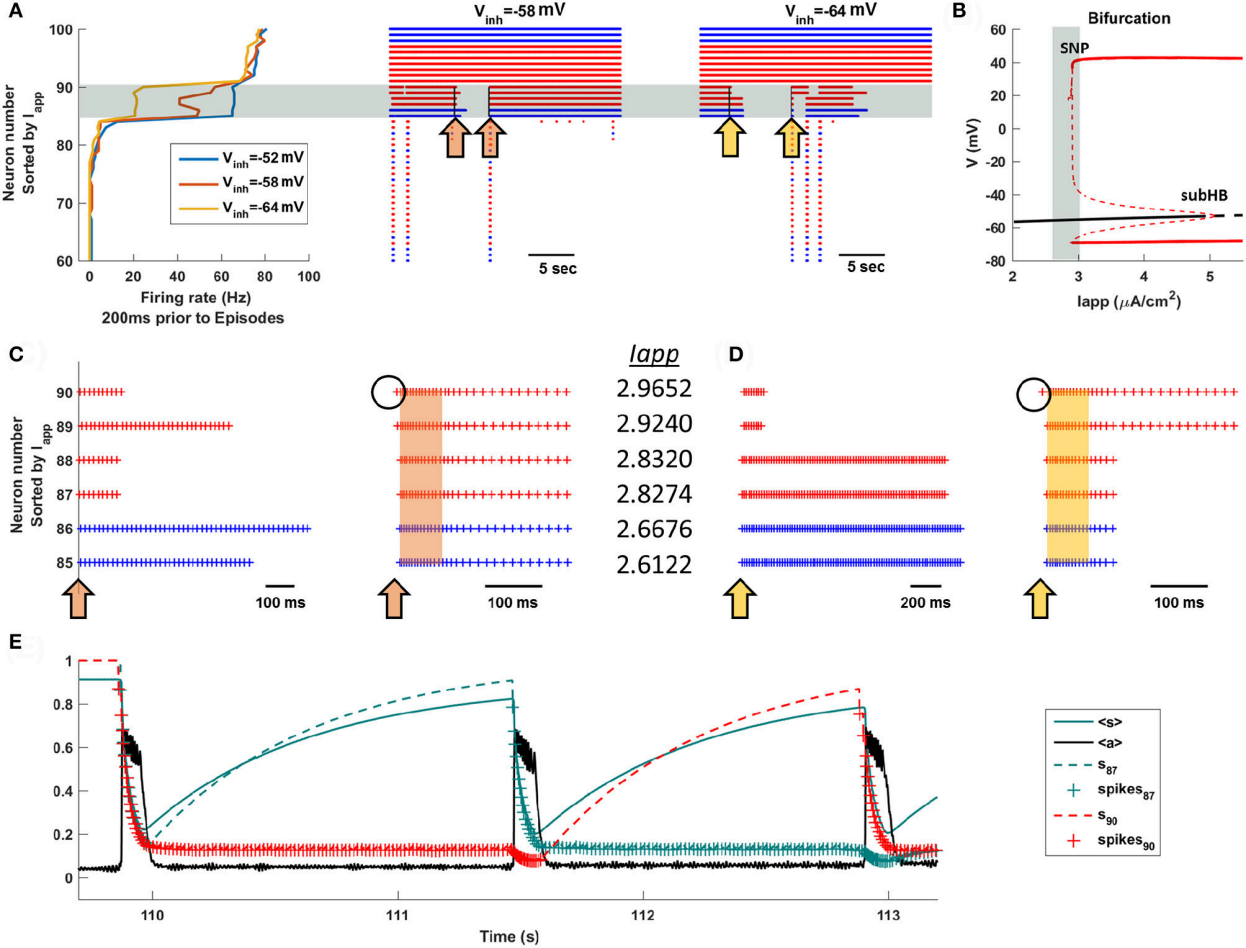
The intermediate population of neurons drives the episodic activity of the entire population. Neurons are sorted according to the value of *I*_*app*_. **(A)** Most neurons do not fire between episodes, and some fire all the time. However, one or more neurons of the intermediate population (in the gray region) fires before an episode, acting as a trigger. The orange arrows (*V*_*inh*_ = −58 mV) in the raster plot indicate time points in which the intermediate neurons begin to turn off, inducing priming, or turn on, triggering a new episode. The yellow arrows have a similar interpretation, but with *V*_*inh*_ = −64 mV. **(B)** Bifurcation diagram for a single uncoupled neuron shows that the intermediate neurons (gray region) have *I*_*app*_ values near the start of the tonic spiking branch. The diagram shows the stationary branch (black) and periodic branch (red), with stable portions indicated by a solid curve and unstable portions by a dashed curve. SNP, saddle node of periodics bifurcation; subHB, subcritical Hopf bifurcation. **(C,D)** Blowup raster plots highlighting the activity of the intermediate neurons. Episodes are indicated by orange and yellow bands. **(E)** Time courses of the efficacy variables, *s*, for neurons 87 (dashed green) and 90 (dashed red), along with the mean efficacy, <*s*> (solid green), and the mean activity, <*a*> (black), with *V*_*inh*_ = −64 mV. In the *s* traces, plus signs correspond to a spiking neuron.

What is so special about the intermediate neurons? What makes them so important for episode triggering and priming? We examine these questions in Figure 5, where we reorder the 100 neurons of the total population according to the size of the applied current that they receive. That is, those with the smallest (most negative) values of *I*_*app*_ are at the bottom of the ordering, while those with the largest values (most positive) are at the top. With this ordering, we first investigate which neurons are active immediately prior to an episode. Figure 5A shows the firing rates of the top 40 neurons of the population during the 200 ms prior to the start of episodes. For three different values of *V*_*inh*_, corresponding to values used Figure 4, there is a clear distinction among subpopulations. Most neurons have firing rates near 0 (they don't fire prior to the start of an episode), the top 10 neurons fire at a high rate (they fire tonically before the episode), and a narrow band of six neurons (in gray) fire at an intermediate rate during the 200 ms prior to episodes. This reflects both a lower frequency of tonic firing and the fact that the neurons are sometimes firing and sometimes not. Importantly, the intermediate neurons are the same subpopulation for each value of *V*_*inh*_ shown. When the GABAergic neurons are more inhibitory these intermediate neurons, but none of the others, fire at a lower average rate in the 200 ms leading up to episodes. Raster plots covering several episodes and inter-episodes point out that these are also the neurons that fire for some time after episodes, but often turn off at some point during the IEI.

Why do the intermediate neurons behave so differently from the others? To understand this, we next examine the single-neuron (uncoupled) behavior as the applied current is varied. Recall that all 100 neurons are identical except for the parameter *I*_*app*_. Figure 5B shows a bifurcation diagram, illustrating both steady states (black curves) and periodic (tonic spiking) solutions. When *I*_*app*_ is low there is a single stable steady state, so neurons with these values of *I*_*app*_ will be silent unless other neurons of the intact network provide the synaptic input necessary to bring them above the threshold for spiking. The branch of steady states loses stability at *I*_*app*_ ≈ 5 μA/cm^2^ at a subcritical Hopf bifurcation (subHB), and gives rise to a branch of unstable periodic solutions. This branch switches back and gains stability at a saddle node of periodics (SNP) bifurcation, beyond which there are stable periodic solutions corresponding to tonic spiking. (see Bertram, 2015 for a description of subHB and SNP bifurcations). In the parameter interval between the SNP and the subHB the system is bistable, although the basin of attraction of the periodic solutions is much larger. In brief, neurons to the left of the gray vertical band in Figure 5B are silent unless pushed above the spike threshold by other neurons in the intact network, those to the right of the vertical band exhibit tonic spiking, while those in the gray band are near or past the SNP and can be either silent or spiking. These latter neurons are the intermediate neurons.

The middle and right panels of Figure 5A (corresponding to *V*_*inh*_ = −58 mV and *V*_*inh*_ = −64 mV, respectively) show how the intermediate neurons control the network behavior. First, they often turn off during IEIs, extending past one episode but not all the way to the next (left arrows in blow up Figures 5C,D). By turning off, they become primed so that when they turn back on they have a strong influence on the other neurons of the network. Thus, the intermediate neurons are special; they can turn back on and spike tonically since they are near the threshold for tonic spiking (the SNP bifurcation). Because of priming, once one of these neurons begins to fire it influences other intermediate neurons to fire (right arrows in Figures 5C,D), which very quickly recruits all neurons in the population to a firing state that characterizes an episode.

Figure 5E shows the time courses of the synaptic efficacy, *s*, for two different intermediate neurons, along with the mean efficacy, <*s*> (solid green curve), superimposed on the network activity profile (black curve). By examining the individual *s* values, rather than the population mean, we see the large change that occurs in the efficacy of a neuron when it goes from a firing to a silent state. The dashed green curve is the efficacy for neuron 87. Prior to the first episode it is silent, so *s* ≈ 1. Once the episode begins (*t* ≈ 109.5 s) the neuron is recruited and fires throughout the episode, during which time its efficacy drops so that *s* ≈ 0.2 by the end of the episode. This neuron stops firing soon after the episode is over, and its efficacy slowly rises, dictated by the rate constant α_*s*_. Neuron 90 (dashed red trace), in contrast, continues to fire long after the episode is over, so that its efficacy remains low (spiking is indicated by plus signs on the curve). The second episode is triggered when neuron 87 begins to fire (*t* ≈ 111.5 s). Because it has high efficacy, the spiking of neuron 87 has a much greater impact on the network than does the spiking of neuron 90. During this second episode the efficacy for both neurons declines, and after the episode terminates, it is neuron 87 that continues to spike. The efficacy for neuron 90 now grows, and when this neuron later begins to spike it initiates the third episode (*t* ≈ 112.8 s). After this episode both neurons continue spiking for the remainder of the time shown, so their efficacies remain low. We see from this that any intermediate neuron can initiate an episode, as long as it is primed to do so.

### The occurrence of episodes is irregular when GABAergic synapses are inhibitory

**Figure 6:**
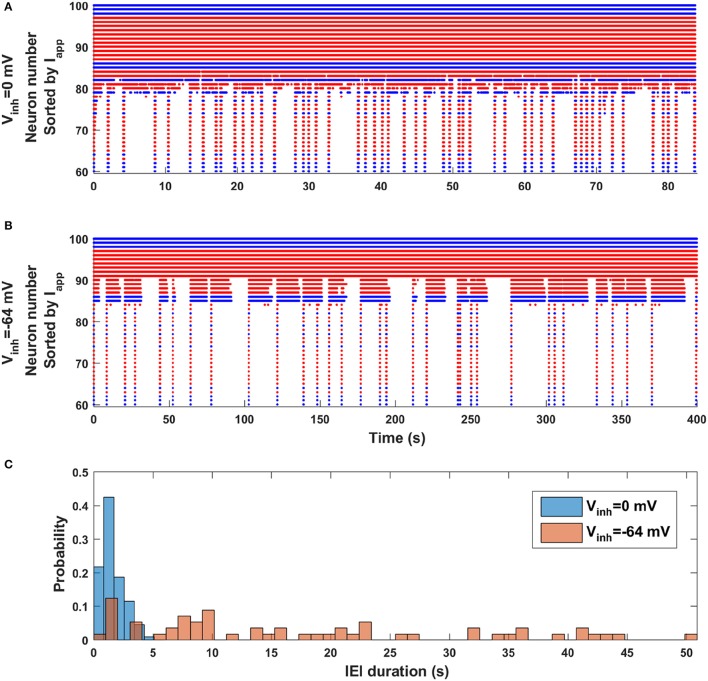
Change in episode regularity as GABAergic synapses switch from excitatory to inhibitory in the neural network model. **(A)** The activity raster plot for the case of excitatory GABAergic synapses (*V*_*inh*_ = 0 mV) shows many short IEIs with a few longer IEI. **(B)** The raster plot for inhibitory GABAergic synapses (*V*_*inh*_ = −64 mV) displays a wide range of IEI durations, with little pattern. **(C)** Histograms of IEI durations for excitatory (blue) and inhibitory (orange) GABAergic synapses indicate a great deal of regularity in the occurrence of episodes when the synapses are excitatory, but little or no regularity when the synapses are inhibitory.

Finally, we revisit the observation made with the mean field model (Figure 2) that episode occurrence is more regular when the synaptic coupling is stronger (*dw* = 0) than when it is weaker (*dw* = 0.17). Is the same true in the network model when the polarity of GABAergic synapses is changed from excitatory to inhibitory? Figure 6 shows two raster plots, the top one corresponding to excitatory GABAergic synapses (*V*_*inh*_ = 0 mV) and the bottom one corresponding to inhibitory GABAergic synapses (*V*_*inh*_ = −64 mV). Although these have very different simulation durations (the bottom one is much longer), by viewing them in this way it appears that the top simulation is dominated by short IEIs with a few longer ones, while the bottom simulation appears to have no dominant IEI duration; there appear to be just as many long-duration IEIs as short-duration IEIs. This observation is quantified in Figure 6C, where IEI duration histograms for the two cases are shown superimposed. When the GABAergic synapses are excitatory the occurrence of an episode is mostly regular, with a strong peak in the IEI duration histogram centered at 1 s (and coefficient of variation of 0.6). In contrast, when the GABAergic synapses are inhibitory the IEI duration distribution is spread out, with no obvious peak (and larger CV of 0.8). Thus, the general regularity of episode occurrence is lost as the polarity of the GABAergic synapses transitions from excitatory to inhibitory. Since this polarity change happens across development, the model makes a very testable prediction about episodic activity in developing neural networks (discussed below).

## Discussion

We have used a computational approach to address the question of how a developmental change in the polarity of GABAergic (and glycinergic) synapses from excitatory to inhibitory affects the pattern of spontaneous activity in a network of neurons with all-to-all coupling. This work builds on experimental observations that many neural systems exhibit episodes of activity early in development (O'Donovan, 1999) when synaptic connections are primarily excitatory (O'Donovan, 1999; Ben-Ari et al., 2007), and that later in development the GABAergic synapses transition from excitatory to inhibitory (Ben-Ari et al., 2007). (But see Bregestovski and Bernard, 2012 for an alternate viewpoint). Because the shift in Cl^−^ reversal potential is driven by activity (Ganguly et al., 2001), it is expected to continue until synaptic inhibition just balances synaptic excitatory connectivity. Some of the changes in network behavior that we observed in response to the GABAergic synapse polarity change were not surprising, and agree with simulations performed with a very simple mean field model (Figures 1, 2). Thus, as the GABAergic synapses become more inhibitory the duration of episodes declines and the duration of inter-episodes increases up to a point (Figure 3). To our surprise, and contrary to the mean field model, once the GABAergic synapse reversal potential reaches a critical point, the neural network model showed that the mean inter-episode duration actually declines as the reversal potential is further reduced. Thus, at this stage of development the mean inter-episode duration begins to get shorter, even though the GABAergic synapses are becoming ever more inhibitory. Similar behaviors were observed with larger networks of 200 and 300 interconnected neurons (see Supplementary Material). We found that this paradoxical behavior is due to a priming effect (Figure 4), which we predict is a ubiquitous phenomenon that would be found in any synapses that exhibit pre- or post-synaptic depression. Priming is particularly important for the class of neurons called intermediate neurons (Vladimirski et al., 2008) which fire action potentials during network activity episodes and at some times between episodes.

Neurons of intermediate excitability play a critical role in triggering episodes in a fully excitatory population, i.e., before we start decreasing *V*_*inh*_. This is because they have (1) a sufficiently low firing rate during the IEI, so their synapses are not as depressed as the synapses of the most excitable neurons in the population; and (2) they are nevertheless very close to threshold compared to the least excitable neurons in the populations (Tsodyks et al., 2000). These neurons comprise a small fraction of the population, and they are the first ones to increase their activity just before an episode (Wiedemann and Lüthi, 2003). Removing part of this population from the network can abolish spontaneous network episodes (Tsodyks et al., 2000; Vladimirski et al., 2008).

Here, we have shown a novel way to define an intermediate population: as *V*_*inh*_ is decreased, the intermediate neurons are the only cells to see their firing rate 200 ms before an episode decrease accordingly (Figure 5A). All other cells show very little change in firing rate as *V*_*inh*_ is decreased, either because they spike spontaneously in the absence of synaptic inputs, or because they do not spike during the IEI. Thus, this intermediate population is also the one responsible for the non-intuitive decrease in IEI at more negative *V*_*inh*_, through the priming mechanism.

According to the priming mechanism, a neuron has to suddenly stop spiking during the interval, so its synapses may recover to a higher value. When the neuron subsequently starts spiking again, it might trigger an episode. Why do these neurons switch back and forth, seemingly randomly, between spiking and silent states during the IEI? This is because these neurons are very near the transition point between silent and tonically active states (Figure 5B).

Thus, a single intermediate neuron can trigger an episode. This could be exaggerated in the particular networks used in this work, since for simplicity and to keep the number of neurons low we have assumed all-to-all connectivity. With random connectivity, not all intermediate neurons may be able to recruit enough neurons to trigger an episode, but in a larger network there will be more intermediate neurons so there should still be some neurons that can trigger an episode. Experimental verification for the properties of intermediate neurons in an experimental preparation may be difficult due to the low number of such neurons (~6% of the total population in the networks used here). Nevertheless, it is technically possible to find them if they do exist (neurons that fire at a very low rate during the IEIs) and they could be voltage clamped near rest, then suddenly depolarized, to attempt to trigger a network episode.

Our studies with the mean field and neural network models recapitulate earlier computational (Tabak et al., 2000, 2001, 2010) and experimental (Streit, 1993; Staley et al., 1998; Sernagor and Grzywacz, 1999; Streit et al., 2001; Tabak et al., 2001; Opitz et al., 2002; Rozzo et al., 2002) findings that the spontaneous episodes of activity in developing neural networks often show a linear correlation between the duration of episodes and the duration of inter-episodes that precede them. We also found that, in the mean field, this correlation was lost when the synaptic coupling was sufficiently reduced (Figure 2), and in the neural network models it was lost once the GABAergic synapses were sufficiently inhibitory (Figure 3). This loss of correlation reflects the loss of regularity of episode occurrences as the GABAergic synapses become more inhibitory (Figures 2, 6). Thus, three additional predictions come from this work that should be testable on systems where spontaneous episodic activity is generated: As development progresses, (1) episodes of activity should become less frequent at relatively early stages of development when the Cl^−^ reversal potential E_Cl_ is still depolarizing, but more frequent at later stages when GABAergic synapses have become inhibitory, (2) the correlation between episode duration and preceding inter-episode duration should disappear at these later stages, as (3) episode occurrences transition from fairly regularly-spaced to highly irregular.

Along with the change in Cl^−^ reversal potential considered here, homeostatic changes in synaptic connectivity and intrinsic cellular excitability (Gonzalez-Islas and Wenner, 2006; Wilhelm et al., 2009; Wenner, 2014) drive network maturation. These homeostatic changes (Gjorgjieva et al., 2016) together with inputs from other areas will lead to a mature, functional network. These changes might mask or potentiate the increase in episode frequency we found when inhibitory synapses have truly become inhibitory. As an example, layer 2/3 cells of the visual cortex of mice exhibit population bursts in early postnatal animals prior to eye opening, with >75% of the neurons firing together in episodes. Immediately after eye opening the episodes become much more frequent and sparser, with <40% firing together in episodes (Rochefort et al., 2009). These dramatic changes are almost certainly due to factors other than changes in GABAergic synapse polarity, such as the introduction of a new input from the retina. In fact, in another study performed in rat cortical slices, population bursts were observed to become less frequent, and disappear altogether in a postnatal time period over which GABAergic synapses transitioned from excitatory to inhibitory (Garaschuk et al., 2000). It will be important in future work to evaluate how each developmental change transform network properties by themselves and when combined together.

Assuming the increase in episode frequency as inhibitory synapses become even more inhibitory can be observed, what possible role could it have? Many of the developmental changes that occur during network maturation, including the change in reversal potential of GABAergic synapses (Ganguly et al., 2001; Garcia-Bereguiain et al., 2016) are dependent on spontaneous activity, which recruits large parts of the network in a coordinated manner. If spontaneous activity stopped too early, this could prevent some of the activity-dependent changes to occur. Thus, the increase in episode frequency as inhibitory synapses become more inhibitory could contribute in maintaining spontaneous, coherent, network activity until a more mature stage of development.

## Author contributions

WB contributed to the study design, computer simulations, and writing of the article. RB contributed to the study design, computer simulations, and writing of the article. JT contributed to the study design, computer simulations, and writing of the article.

### Conflict of interest statement

The authors declare that the research was conducted in the absence of any commercial or financial relationships that could be construed as a potential conflict of interest.
