# Supplementary material for: The Effects of GABAergic Polarity Changes on Episodic Neural Network Activity in Developing Neural Systems
Source: Front Comput Neurosci. 2017 Sep 29;11:88. doi: 10.3389/fncom.2017.00088 (PMC5649201; doi:10.3389/fncom.2017.00088)
Supplement: Supplementary file 1 [file Image1.PDF]

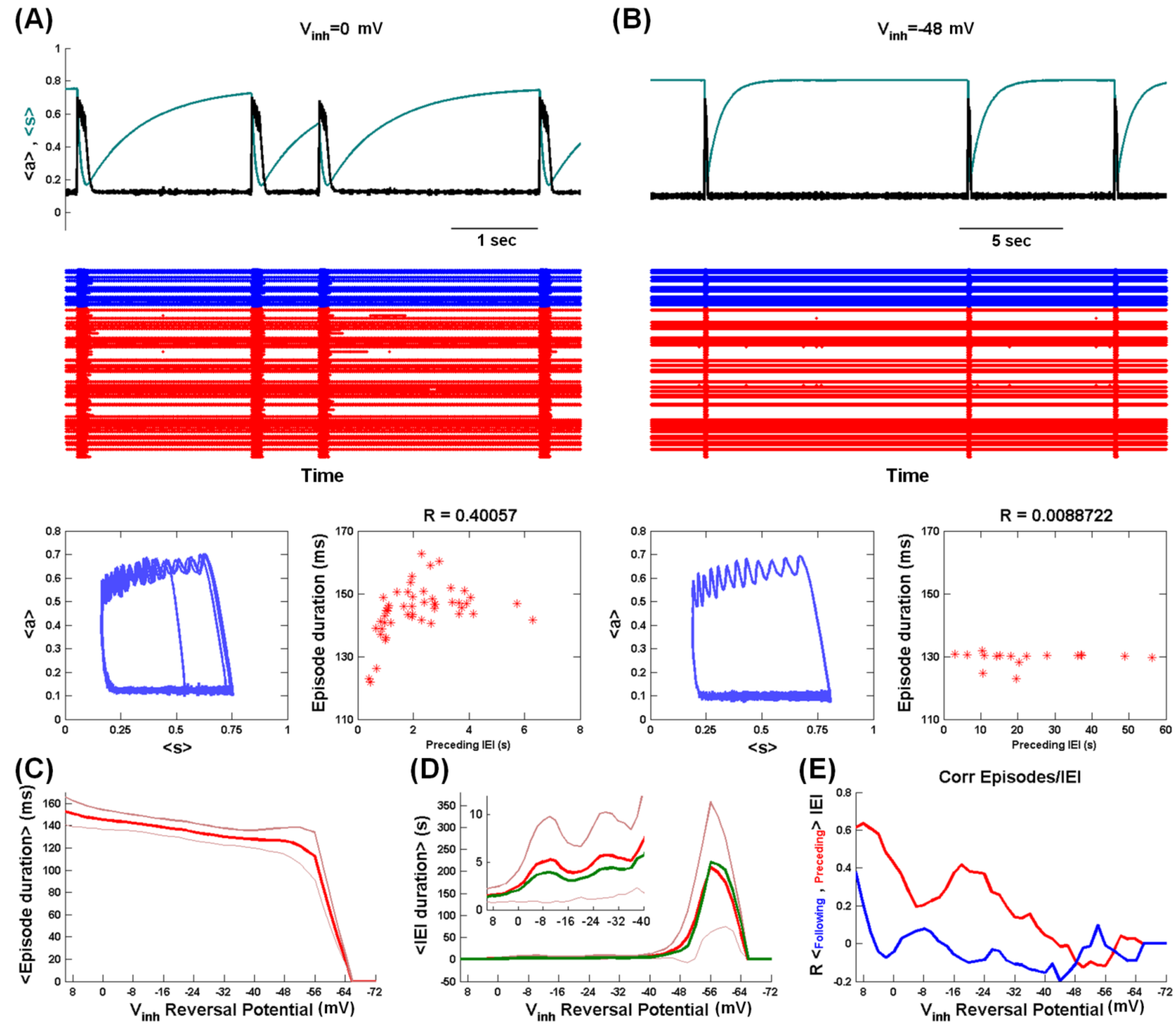

Supplemental Figure 1: Spontaneous episodic activity of the neural network model with 200 neurons, of which 40 are GABAergic, and all-to-all coupling. All features are similar to simulations run with 100 neurons, 20 of which are GABAergic, as shown in Fig. 3 of the manuscript.

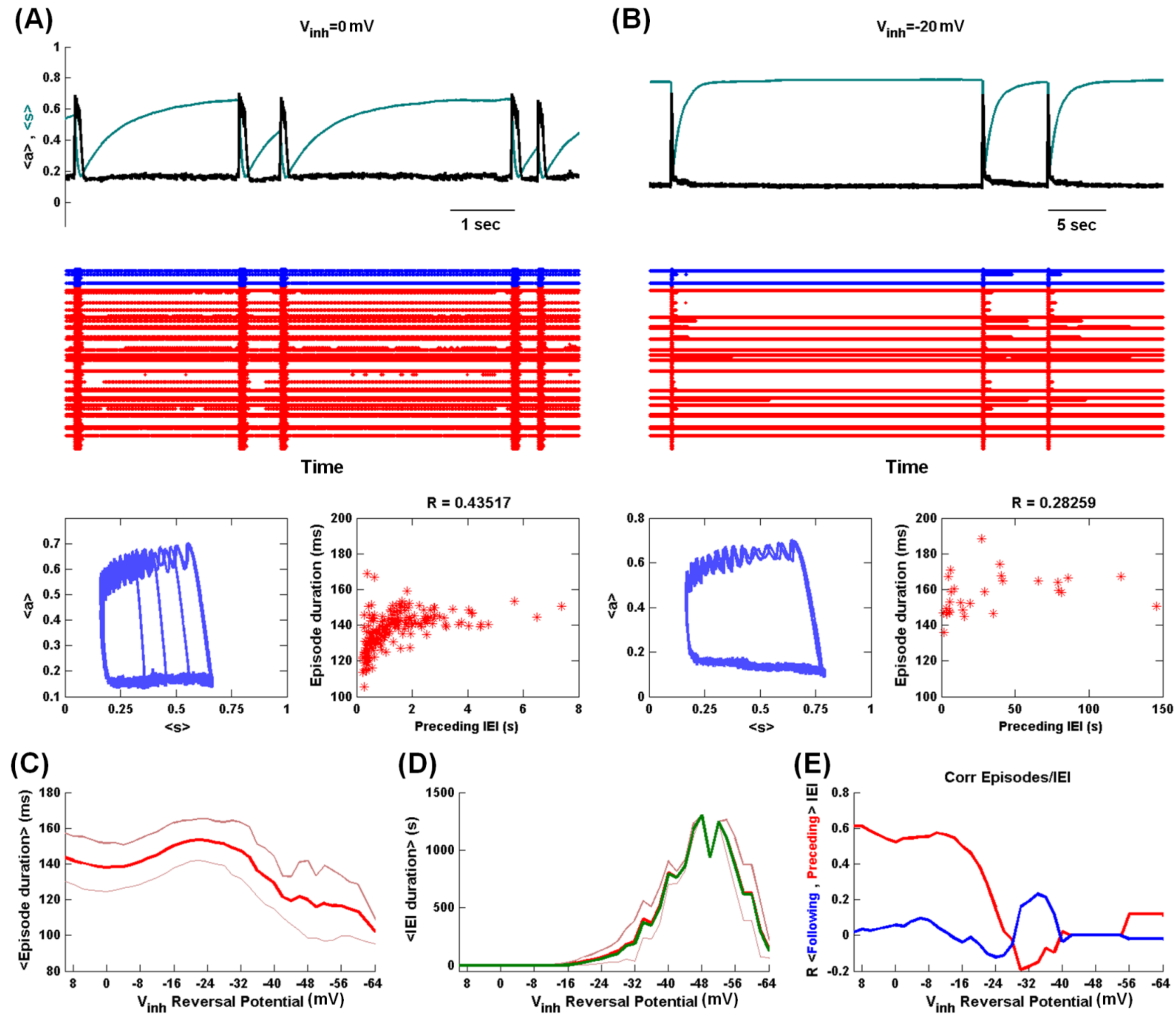

Supplemental Figure 2: Spontaneous episodic activity of the neural network model with 300 neurons, of which 60 are GABAergic, and all-to-all coupling. Increasing the number of neurons does not change the qualitative behavior of the system.
